# The reaction of cucumber to the introduction of ionic liquids into the soil

**DOI:** 10.1007/s11356-020-09686-0

**Published:** 2020-06-16

**Authors:** Robert Biczak, Barbara Pawłowska, Cezary Podsiadło, Martyna Śnioszek, Arkadiusz Telesiński

**Affiliations:** 1 The Faculty of Science and Technology, Jan Długosz University in Czestochowa, 13/15 Armii Krajowej Av., 42-200 Częstochowa, Poland; 2 The Faculty of Environmental, Management and Agriculture, West Pomeranian University of Technology in Szczecin, Juliusza Słowackiego St. 17, 71-434 Szczecin, Poland

**Keywords:** Ionic liquids, Cucumber, Phytotoxicity, Oxidative stress, Antioxidant enzyme activity

## Abstract

This paper presents the influence of two bromides, tetrabutylammonium and tetrabutylphosphonium, on the growth and development of cucumber seedlings. The tests were performed at two dates, i.e. 10 and 20 days, after the introduction of increasing amounts of ionic liquids (ILs) into the soil. The applied ILs showed phytotoxicity dependent mainly on the concentration of the substance, which is proved by the inhibition of the length of aboveground parts and their roots and the yield of cucumber fresh mass, from which EC_50_ values were calculated. The phytotoxicity symptoms were the result of oxidative stress, one of the manifestations of which was a decrease in assimilative pigments, linearly correlated with an increase in bromide concentration in the medium. The stress is also proven by the large increase in hydrogen peroxide, malondialdehyde and free proline in cucumber leaves. The reaction of this plant to oxidative stress was an increase in the activity of antioxidative enzymes such as catalase and peroxidase. As a result of statistical analysis, it was proved that all changes of biomarkers of phytotoxicity of examined ILs and oxidative stress indicators in cucumber seedlings depended more on the applied concentration of these salts than on the date of the study.

## Introduction

Both the size and quality of crops, including vegetables, depend on many factors related to the growing conditions, such as the availability of water, an adequate supply of nutrients and microelements and optimal temperature and lighting. These are factors that limit the growth and development of plants, but the success of vegetable production also depends on the quality of the soil and on the environmental conditions in which the plants grow. A factor limiting the size of the crop, and often also affecting its quality, are all kinds of contamination and pollution of soils, as well as underground and surface waters. This includes soil salinization, the presence in soils and waters of heavy metals, pesticides and their residues and other chemical compounds introduced intentionally or accidentally into the growing environment (Dresler et al. 2019; Parihar et al. 2015; Pereira et al. 2010; Qi et al. 2015).

The environmental burden can be divided into two basic groups: biotic and abiotic. Biotic stress includes the adverse effects of pathogenic microorganisms, fungi or weeds on crops as competitors to water and nutrients. The sources of abiotic stress include extreme temperatures, droughts, mechanical damage, air pollution, high light levels, salinity or the chemical agents mentioned above. When these stress parameters exceed the limit value, all symptoms of oxidative stress appear very quickly in plants grown in such conditions. The primary symptom of oxidative stress is the overproduction of reactive oxygen species (ROS), represented by superoxide anion, singlet oxygen, hydroxyl radical and hydrogen peroxide. Under homeostatic conditions, when the balance of production and demand for ROS is under strict control, they are very important signal molecules, essential for the proper functioning of plant metabolism. However, under oxidative stress, the overproduction of ROS leads to damage to plant cells through oxidation of nucleic acids, proteins and fats. Physiological and molecular changes at the cellular level include loss of turgidity, lack of membrane fluidity, changes in the concentration of cellular juice contained in the vacuole and reduction of photosynthetic activity, which occurs as a result of stomach apparatus closure, ineffective electron transport and decreased activity of enzymes responsible for the photosynthesis process. All of this leads to a reduction in the growth rate of plants and their productivity, and under extreme stress conditions, it can be fatal for organisms (Bartwal et al. 2013; Kubiś 2008; Mittler 2017).

Therefore, the main trend of science nowadays is, among other things, the search for chemical compounds that will not be a burden on the environment. Intensive research in green chemistry focuses on the design of environmentally friendly solvents to eliminate volatile organic solvents from chemistry. Following these criteria, scientists have drawn attention to a particular type of chemical compound, ionic liquids (ILs), because of their physico-chemical properties. Ionic liquids have been recognized as innovative solvents with a wide range of potential applications, and their properties such as low vapour pressure, non-flammability, non-volatility, high ionic conductivity and thermal and chemical stability are decisive. In addition, even unlimited possibilities of selection and exchange of anions and cations make it possible to obtain ionic liquids with predetermined and strictly controlled properties such as hydrophobicity, viscosity, density, solubility, biodegradability or toxicity. Due to these properties, ILs can be used in many areas of industry and agriculture (Egorova and Ananikov 2014, 2018; Isosaari et al. 2019; Montalbán et al. 2018; Tot et al. 2018a; Vraneš et al. 2018).

However, due to the high thermal and chemical stability of ILs, so desirable in many chemical processes, these compounds can become persistent contaminants in the environment. Therefore, before using ILs on an industrial scale, it is essential that potential environmental risks are carefully assessed by determining the persistence of ILs in water and soil, migration and accumulation in groundwater and surface water, accumulation in organisms and overall ecotoxicity of these substances. Unfortunately, the ecotoxicological studies carried out on ILs have led to the questioning of the appropriateness of using the term “green solvents” for ILs, because the toxicity of these compounds for a number of organisms, i.e. bacteria, fungi, algae, plankton, higher plants, invertebrates and vertebrates, was undeniably demonstrated. Many works explain the toxic effects of ILs on organisms caused by these compounds to induce oxidative stress, resulting in an increase in the concentration of ROS, non-enzymatic antioxidants and changes in the activity of antioxidative enzymes observed in cells (Biczak 2017; Cvjetko Bubalo et al. 2014; Liu et al. 2016a, 2017, 2018a; Pawłowska et al. 2019a; Xu et al. 2020).

In the presented studies, the effect of two ILs—tetrabutylammonium and tetrabutylphosphonium bromide—introduced into the soil on the growth and development of cucumber seedlings (*Cucumis sativus* L.) was determined. An attempt was also made to determine how the effect of the ionic liquids tested on plants during the experiment was changed. Due to its high yields and many nutrients, the cucumber is a vegetable grown on a very large scale all over the world. Unfortunately, the relatively shallow root system of the plant and limited regeneration possibilities make cucumbers very susceptible not only to water shortage but also to soil pollution (Liu et al. 2009; Tang et al. 2018; Zhang et al. 2007). According to our knowledge, there is currently only one work in the available literature (Tot et al. 2018b) in which the authors have undertaken the task of determining the effect of ionic liquids on the germination of cucumber seeds and the growth and development of early stages of this vegetable. The research described by the authors also concerns the hydroponic cultivation of a vegetable which, although it limits the influence of factors other than ILs on the plant, is not able to replace and show all the dependencies and mechanisms of influence that are characteristic for the soil environment in which this vegetable is grown naturally.

Tetrabutylammonium bromide and tetrabutylphosphonium bromide were selected for the study. These compounds are used as solvents and catalysts in various chemical reactions. From the available knowledge, there are currently no studies attempting to assess and compare the effect of tetrabutylammonium bromide and tetrabutylphosphonium bromides on cucumbers and how this interaction depends on the length of contact of plants with ILs introduced into the soil. We are therefore convinced that the results of our experiments will fill this gap in the scientific literature and will be useful for developing recommendations for cucumber cultivation.

## Materials and methods

### Materials

The ionic liquids, tetrabutylammonium bromide [TBA][Br] (≥ 98% purity) and tetrabutylphosphonium bromide [TBP][Br] (98% purity), used in the study were purchased from Sigma-Aldrich Chemical Co.

### The vase experiment

Phytotoxicity tests [TBA][Br][Br] and [TBP][Br] have been carried out in the vegetation hall of the Department of Biochemistry, Biotechnology and Ecotoxicology. The tests were carried out according to the guidelines of OECD/OCDE 2006 guide. Ten identical cucumber seeds (*Cucumis sativus* L.) of Octopus F1 cultivar were sown in plastic pots filled with 250 g of control soil (without ILs) and soil with [TBA][Br] and [TBP][Br] at concentrations of 1, 10, 100, 400, 700 and 1000 mg kg^−1^ of soil DW. The soil used in the experiment was clayey sand, with about 11% fraction content of < 0.02 mm in diameter, organic carbon – 8.5 g kg^−1^ and pH(KCl) equal to 6.0. The studied compounds were introduced into the soil as aqueous solutions and thoroughly mixed. Throughout the whole period of the study, constant humidity of the substrate (70% ppw), temperature 20 °C ± 2 °C and lighting at the level of 170 μmol^−2^ s^−1^ in the 16 h day/8 h night system were maintained. In order to perform chemical analyses, plant samples were taken 10 and 20 days after seeding. All analyses were repeated three times.

### Determination of basic phytotoxicity parameters

In order to determine the toxicity of [TBA][Br] and [TBP][Br] for cucumber, the germination potential (GP) and seed germination rate (GR) were determined. Seeds for which the germ was larger than 2 mm were considered to be germinated (Liu et al. 2014).

Inhibition of growth of aboveground parts of plants and their roots was determined according to Wang et al. (2009). The yield of fresh plant mass was also determined. The inhibition factor was calculated from the relation:

$$ \frac{\mathrm{Length}/\mathrm{weight}\ \mathrm{in}\ \mathrm{control}\ \mathrm{group}-\mathrm{length}/\mathrm{weight}\ \mathrm{in}\kern0.5em \mathrm{ILs}\ \mathrm{treated}\ \mathrm{group}\ }{\mathrm{Length}/\mathrm{weight}\ \mathrm{in}\ \mathrm{control}\ \mathrm{group}}\times 100\% $$

The results were expressed as % inhibition of fresh weight yield or length of roots and overground parts. Non-linear regression analysis was used to estimate effective concentrations (EC_50_) using GraphPad Prism software (GraphPad Software, Inc., La Jolla, CA, USA).

The level of dry weight (DW) was determined by the weight-drying method (Kowalska 2004). Approximately 1 g of fresh plant weight was dried at 105 °C to obtain a constant weight. The dry weight content is given in g g^−1^ fresh weight (FW).

### Determination of assimilation pigments content

Five hundred milligrams of fresh leaf mass was homogenized with 80% acetone at 4 °C and then centrifuged at 10000 rpm for 10 min. The absorbance of the supernatant was measured at 470 nm, 647 nm and 664 nm. The assimilation pigments content was determined according to the Oren et al. (1993)’s method. The photosynthetic pigments content was expressed in mg g^−1^ DW.

### Determination of MDA content

The malondialdehyde content (MDA) was determined in accordance with the method described by Hodges et al. (1999). Five hundred milligrams of fresh leaf mass was homogenized with the addition of chilled (4 °C) 0.1% trichloroacetic acid solution and was centrifuged at 10000 rpm for 10 min. The MDA content was determined by absorbance measurements at 532 nm and 600 nm and then calculated using an extinction coefficient equal to 155 nm^−1^ cm^−1^ and expressed in μmol g^−1^ FW.

### Determination of H_2_O_2_ content

The H_2_O_2_ content was determined according to Singh et al. (2007). 500 mg of fresh leaf mass was homogenized with the addition of chilled (4 °C) 0.1% trichloroacetic acid solution was centrifuged at 10000 rpm for 10 min. Absorbances of the mixture (supernatant + phosphate buffer with pH = 7.0 + KI) were measured at 390 nm. The content of H2O2 was calculated using an extinction coefficient equal to 155,155 nm^−1^ ∙ cm^−1^ and expressed in μmol ∙ g^−1^ FW.

### Determination of free proline content

The free proline content was determined according to Bates et al. (1973). Five hundred milligrams of fresh plant sample was homogenized with 5 ml of 3% sulfosalicylic acid. Two millilitre of supernatant was added to a mixture of 2 ml of glacial acetic acid and 2 ml of 2.5% (w/w) of acidic ninhydrin. The reaction was carried out at 100 °C for 1 h, and then the reaction was interrupted in an ice bath. Five millilitre of toluene was added to the solution. Absorbance was measured at 520 nm, and the calculated proline content was given in mg g^−1^ FW.

### Determination of antioxidant enzyme activity

Five hundred milligrams of fresh leaf mass was homogenized with the addition of chilled (4 °C) extraction mixture (phosphate buffer pH = 7.4 + 1 mM EDTA solution + 0.1% polyvinylpyrrolidone (PVP) solution). Homogenate was centrifuged at 10000 rpm for 10 min, and the supernatant obtained was used to determine the activity of the enzymes and protein content.

The activity of superoxide dismutase (SOD) [EC 1.15.1.1] was determined spectrophotometrically by measuring nitrotetrazolium blue (NBT) reduction according to Giannopolitis and Ries (1977). The absorbance of the reaction mixture was measured at 560 nm. SOD activity was expressed in units of activity—U mg^−1^ protein. One unit of SOD activity (U) – the amount of enzyme causing 50% inhibition of NBT reduction reaction rate.

Catalase activity (CAT) [EC 1.11.1.6] was determined by titration according to the method proposed by Kar and Mishra (1976). The CAT activity was determined by the H_2_O_2_ distribution over 15 min. The remaining H_2_O_2_ was titrated with 0.01 N solution of KMnO_4_. Catalase activity was expressed as U mg^−1^ protein min^−1^.

The activity of peroxidase (POD) [EC 1.11.1.7] was determined spectrophotometrically by determining the oxidation rate of guaiacol in the presence of H_2_O_2_ for 1 min at 470 nm (Abbasi et al. 1998). The peroxidase activity was expressed as U mg^−1^ protein min^−1^.

The total protein content (needed to calculate the enzymatic activity of SOD, CAT and POD) was determined by Bradford (1976) method using Coomasine Blue.

### Statistical analysis

In this experiment, all analyses were carried out in three independent repetitions. The obtained results were then statistically analysed using STATISTICA 12.5. The data from three measurements (*n* = 3) were analysed using two-factor and three-factor analysis of variance (ANOVA) using a post hoc test—the Tukey test. The significance level was *p <* 0.05. The results were expressed as mean ± standard deviation. Based on the analysis of the effect measure *η*^2^ by variance analysis, the percentage shares of all variable factors affecting the assayed plant parameters were defined. In order to establish relationships between individual phytotoxicity and oxidative stress parameters in cucumber seedlings under the influence of ILs, the analysis of main components (PCA) was also carried out.

## Results and discussion

### Phytotoxicity assay of ILs

In order to assess the phytotoxicity of the studied ILs, the potential and germination capacity of cucumber seeds, inhibition of root and aboveground growth of this vegetable, fresh weight yield inhibition and dry weight content were determined. On the basis of yield inhibition, length of aboveground parts and roots of cucumber seedlings EC_50_ values were calculated.

The first, and at the same time most important, stage of growth of each plant is germination. In addition to optimum substrate moisture, the presence of various stress factors, both biotic and abiotic, has a huge impact on the germination of crops, which is, therefore, one of the very important problems of modern agriculture. The analysis of the results obtained in the conducted research proves that an increase in the concentration in the soil of the studied bromides led to a systematic decrease in germination potential (GP) values of cucumber seeds. In the case of germination capacity (GR), the differences found were not statistically significant after application of [TBA][Br], while the highest concentrations of [TBP][Br] (700 and 1000 mg kg^−1^ of soil DW) led to a clear inhibition of this parameter in relation to control (Table 1).

**Table 1:** Effect of ILs on the germination potential (GP) and germination rate (GR) of cucumber

Concentration of ILs (mg kg^−1^ of soil DW)	[TBA][Br]		[TBP][Br]	
	GP (%)	GR (%)	GP (%)	GR (%)
0	82.50 ± 9.57^a^	90.00 ± 8.16^a^	75.00 ± 19.15^a^	88.00 ± 14.14^abc^
1	75.00 ± 5.77^ab^	85.00 ± 5.77^a^	80.00 ± 8.16^a^	90.00 ± 8.16^a^
10	77.50 ± 12.58^ab^	87.50 ± 5.00^a^	80.00 ± 8.16^a^	87.50 ± 9.57^ab^
100	57.50 ± 9.57^abc^	77.50 ± 7.08^a^	72.50 ± 15.00^a^	90.00 ± 8.16^a^
400	52.50 ± 12.58^bcd^	82.50 ± 9.57^a^	57.50 ± 5.00^ab^	87.50 ± 5.00^ab^
700	35.00 ± 9.15^cd^	85.00 ± 17.32^a^	30.00 ± 14.14^b^	65.00 ± 12.91^bc^
1000	30.00 ± 8.16^d^	77.50 ± 5.00^a^	30.00 ± 14.14^b^	62.50 ± 9.57^c^

Data are means ± SD from 3 independent experiments. Values denoted by the same letters in the columns do not differ statistically at *p <* 0.05

Tot et al. (2018b) obtained similar results in his research, in which he determined the effect of imidazole ionic liquids differing in substituent length and type of anion on cucumber seed germination. The authors quoted stated a clear inhibition of seed germination, which was correlated with increasing concentration of examined ILs in the medium. Other authors (Liu et al. 2014; Cvjetko Bubalo et al. 2014; Pawłowska et al. 2019b; Tot et al. 2018a; Vraneš et al. 2018) have also reached similar conclusions in their studies on the effect of ionic liquids on the germination power of various higher plant species. However, there are papers (Biczak 2017; Biczak et al. 2017) in which the authors did not find any significant influence of ILs on the germination capacity and potential of seeds, which allows concluding that these phytotoxicity parameters are highly dependent on the plant species. The type of compound is also important here, especially the concentration used.

**Fig. 1:**
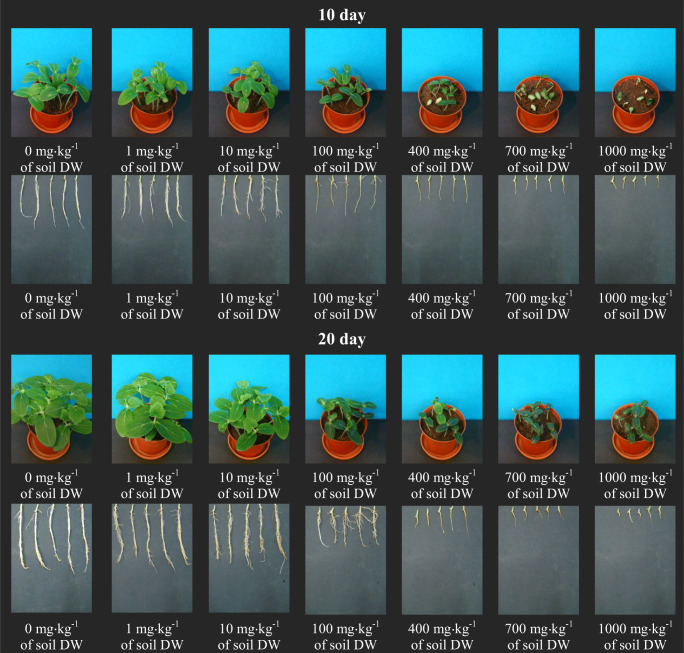
Digital photographs of cucumber seedlings and roots on the 10th and 20th day after introduction to the soil [TBA][Br]

**Fig. 2:**
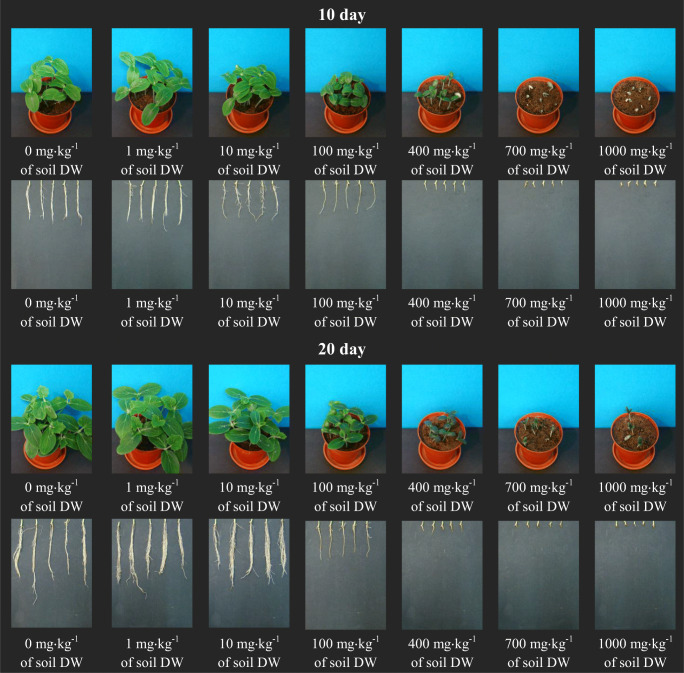
Digital photographs of cucumber seedlings and roots on the 10th and 20th day after introduction to the soil [TBP][Br]

The visible reduction in the potential and germination capacity of cucumber seeds had a clear impact on further growth and development of seedlings of this plant. Both [TBA][Br] and [TBP][Br] caused a clear inhibition of root and shoot length of spring barley seedlings. After applying higher concentrations (400–1000 mg kg^−1^ of soil DW) of both ILs, several seeds germinated, and the plants that grew were dwarfish with clear spots indicating leaf chlorosis. On the basis of the appearance of plants, one can risk an opinion that the ionic liquid containing a phosphorus atom in its structure had a more unfavourable effect on the growth and development of cucumber (Figs. 1 and 2).

**Fig. 3:**
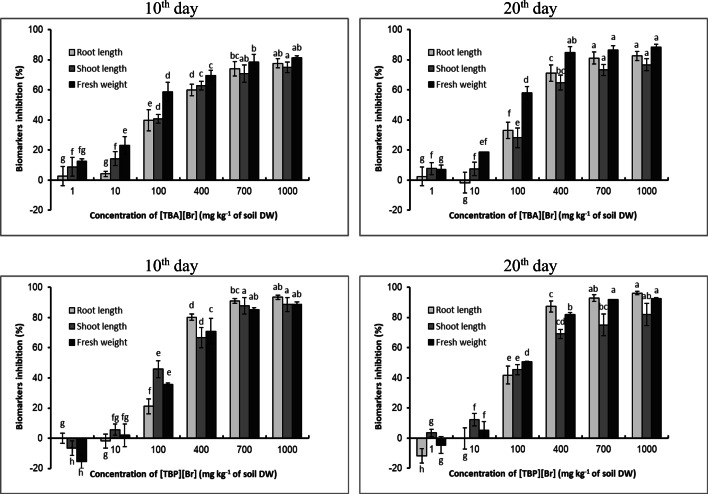
The inhibition rate (%) of root length, shoot length and fresh weight of seedlings of cucumber after exposure to [TBA][Br] and [TBP][Br]. Data are means ± SD from 3 independent experiments. Values denoted by the same letters for the same biomarkers do not differ statistically at *p* < 0.05

The digital photos presented in Figs. 1 and 2 also indicate that the bromides used in this experiment clearly influenced the growth and development of the root system of cucumber seedlings. Starting from a concentration of 400 mg kg^−1^ of soil DW, both ionic liquids led to a situation where it can be concluded that the cucumber root system has not developed at all. What can be seen in the pictures are only root bundles, completely devoid of side roots. This was especially visible after the introduction of [TBP][Br]. This clear reaction of cucumber seedling roots to the presence of ILs in the soil is due to the fact that the roots are the first organ to come into direct and continuous contact with impurities present in the soil. This can cause damage to the cell membrane of the roots, resulting in toxins penetrating the roots and spreading them further throughout the plant organism (Chapman et al. 2012). Due to their functions, i.e. maintaining the plant in the substrate, taking up water and nutrients and transporting them, the roots are very important organs of the plant, and their proper development and condition determine the optimal growth and development of the plant, which in turn translates into the size and quality of the yield. Therefore, the impairment of the root system causes disturbances in the development of the whole plant and, as a result, inhibits the length of the aboveground parts and the yield of fresh plant mass. In the experiment in question, a clear reduction in the length of aboveground parts of cucumber seedlings occurred after applying both ILs at a concentration of 100 mg kg^−1^ of soil DW. This in turn was reflected in the inhibition of the yield of fresh plant mass, which was positively correlated with the increase in ILs concentration in the medium. At the same time, it should be noted that in the second period of analysis, a slightly greater inhibition of the length of aboveground parts and fresh mass of cucumber seedlings was observed, which was certainly a consequence of underdevelopment of the plant root system (Figs. 1,2 and 3).

These results are confirmed by previous literature reports (Chen et al. 2018; Cvjetko Bubalo et al. 2014; Fan et al. 2019; Liu et al. 2015; Yu et al. 2018; Pawłowska et al. 2019b; Tot et al. 2018 a and b; Vraneš et al. 2018; Xu et al. 2018). These authors prove that inhibition of root and shoot length is one of the most obvious symptoms of phytotoxicity in plants. Moreover, these authors agree that low IL concentrations can stimulate plant growth, while higher ILs concentrations inhibit plant growth and fresh weight yield. However, there are reports that plant resistance to ILs is highly dependent on the genetic characteristics of plant species and varieties (Biczak et al. 2014; Liu et al. 2018a). The calculated percentages of root length inhibition, the length of aboveground parts and the yield of fresh plant mass allowed to determine EC_50_ (Table 2).

**Table 2:** The EC_50_ values and 95% confidence intervals for cucumber seedlings following exposure to ILs

	10th day	20th day
[TBA][Br]		
Inhibition for fresh weight	57.18 ± 5.96 (11.37–287.5)	68.07 ± 3.02 (34.64–133.7)
Inhibition for root length	155.3 ± 5.5	141.6 ± 2,4 (71.80–279.1)
Inhibition for shoot length	162.1 ± 0.8 (106.0–248.0)	181.2 ± 0.4 (156.7–209.4)
[TBP][Br]		
Inhibition for fresh weight	77.6 ± 10.0	76.44 ± 6.41 (23.79–245.6)
Inhibition for root length	161.9 ± 0.9 (136.3–209.7)	114.1 ± 4.1 (49.70–261.9)
Inhibition for shoot length	136.3 ± 13.6	123.0 ± 2.5 (48.33–313.2)

The analysis of the obtained EC_50_ values confirms the observations made earlier on the basis of the external appearance of cucumber plants and the calculated percentage inhibition values of root length, overground parts and fresh weight yield. Furthermore, no clear differences in EC_50_ values were found for the two test dates. On the one hand, this may result from the fact that the cucumber plants have not yet managed to activate the protective mechanisms against toxic effects of the examined ILs, and on the other hand, it may be a consequence of the lack of absorption of these compounds on soil colloids. As Stepnowski et al. (2007) write, only compounds with long substituents are retained relatively quickly by soil organic matter.

A very important biomarker, indicating the toxicity of chemical compounds, are the changes in the level of the dry weight of plants; therefore, this parameter is often examined in works on phytotoxicity (Biczak et al. 2014, 2017; Chen et al. 2018; Pawłowska et al. 2019b). The quoted studies prove a progressive increase in the level of dry weight in plants cultivated on soil with ILs content. The presented studies also showed a linear increase in dry weight content in cucumber seedlings, which at the highest concentrations of bromide salts exceeded 100% compared with the control. However, no statistically proven differences in dry weight content were found in the subsequent test periods (Table 3).

**Table 3:** Effect of ILs on the content of dry weight in seedlings of cucumber

Concentration of ILs (mg kg^−1^ of soil DW)	Dry weight (g g^−1^ FW)	
	[TBA][Br]	[TBP][Br]
10th day		
0	0.0711 ± 0.0012^f^	0.0724 ± 0.0025^g^
1	0.0726 ± 0.0020^ef^	0.0730 ± 0.0008^g^
10	0.0774 ± 0.0024^ef^	0.0788 ± 0.0024^fg^
100	0.0989 ± 0.0016^d^	0.1003 ± 0.0017^e^
400	0.1331 ± 0.0030^c^	0.1340 ± 0.0050^d^
700	0.1793 ± 0.0040^b^	0.1735 ± 0.0063^bc^
1000	0.1971 ± 0.0015^a^	0.1948 ± 0.0049^a^
20th day		
0	0.0891 ± 0.0083^de^	0.0710 ± 0.0048^g^
1	0.0956 ± 0.0100^d^	0.0730 ± 0.0047^g^
10	0.0876 ± 0.0040^def^	0.0784 ± 0.0069^fg^
100	0.0903 ± 0.0058^de^	0.0930 ± 0.0022^ef^
400	0.1273 ± 0.0023^c^	0.1364 ± 0.0032^d^
700	0.1771 ± 0.0090^b^	0.1684 ± 0.0103^c^
1000	0.1984 ± 0.0123^a^	0.1840 ± 0.0051^ab^

Data are means ± SD from 3 independent experiments. Values denoted by the same letters in the columns do not differ statistically at *p <* 0.05

This trend in plant dry weight changes is related to the fact that high IL concentrations can lead to symptoms of typical soil salinity, which in turn makes it more difficult for plants to absorb water and limits its availability. As a result, the turgidity of plant cells decreases and leads to the observed accumulation of dry weight in cucumber seedlings.

### Effect of ILs on pigments content

The primary production of plants depends on the efficiency of the photosynthesis process, which is influenced by both the amount of assimilative pigments and their mutual ratio. By converting light energy into chemical energy, chlorophylls are very vulnerable to ROS overproduction. When the antioxidant defence system fails, excited chlorophyll reacts with oxygen to give a range of free oxygen radicals, leading to protein damage in PSII and reduced photosynthesis efficiency. Therefore, in studies on the determination of the abiotic and biotic effects of oxidative stress factors, the determination of assimilation pigments has become the norm. In the scientific literature describing the influence of ILs on plant growth and development, there are also studies in which the influence of these compounds on the content of assimilation pigments is sometimes considered to be the most important biomarker of the occurring oxidative stress. The majority of these studies prove that ILs are compounds that interfere with normal metabolism and cause changes in cell structures, including damage to photosynthetic pigments, and influence the fluorescence parameters of chlorophyll. The authors report an even linear decrease in assimilation pigments content in plants with an increase in ILs medium (Biczak 2017; Chen et al. 2018, 2019; Deng et al. 2017; Liu et al. 2014, 2015, 2016a, 2018b; Wang et al. 2009; Xia et al. 2018; Xu et al. 2018).

In the presented studies on the influence of [TBA][Br][Br] and [TBP][Br] on the growth and development of early stages of cucumber, also changes in the level of all assimilation pigments were determined (Tables 4 and 5).

**Table 4:** Effect of [TBA][Br] on the photosynthetic pigment in seedlings of cucumber

Concentration of [TBA][Br] (m kg^−1^ of soil DW)	Pigments (mg g^−1^ DW)					
	Chl*a*	Chl*b*	Chl*a* + Chl*b*	Chl*a*/Chl*b*	Car	Chl(*a* + *b*)/Car
10th day						
0	16.646 ± 0.063^c^	4.741 ± 0.031^b^	21.387 ± 0.068^c^	3.511 ± 0.028^cd^	4.005 ± 0.021^c^	5.340 ± 0.029^h^
1	16.171 ± 0.095^e^	4.617 ± 0.046^c^	20.788 ± 0.117^d^	3.503 ± 0.035^cd^	3.900 ± 0.022^e^	5.330 ± 0.026^h^
10	16.416 ± 0.008^d^	4.612 ± 0.046^c^	21.029 ± 0.049^d^	3.559 ± 0.035^bc^	3.937 ± 0.025^de^	5.341 ± 0.044^h^
100	17.157 ± 0.025^b^	4.824 ± 0.040^ab^	21.981 ± 0.065^b^	3.557 ± 0.024^bc^	3.959 ± 0.027^cd^	5.553 ± 0.047^f^
400	12.851 ± 0.013^g^	3.766 ± 0.014^d^	16.617 ± 0.009^f^	3.413 ± 0.016^e^	2.923 ± 0.004^h^	5.685 ± 0.006^e^
700	8.597 ± 0.022^k^	2.439 ± 0.011^g^	11.036 ± 0.033^j^	3.525 ± 0.007^cd^	1.895 ± 0.004^k^	5.825 ± 0.029^d^
1000	8.475 ± 0.026^k^	2.348 ± 0.011^g^	10.823 ± 0.024^j^	3.610 ± 0.023^ab^	1.802 ± 0.005^l^	6.006 ± 0.019^c^
20th day						
0	13.836 ± 0.047^bc^	3.793 ± 0.021^d^	17.630 ± 0.054^e^	3.648 ± 0.022^a^	3.544 ± 0.009^f^	4.975 ± 0.019^j^
1	13.000 ± 0.072^g^	3.736 ± 0.035^d^	16.736 ± 0.103^f^	3.480 ± 0.019^de^	3.150 ± 0.012^g^	5.312 ± 0.025^h^
10	17.221 ± 0.126^b^	4.870 ± 0.052^c^	22.090 ± 0.210^b^	3.537 ± 0.029^cd^	4.072 ± 0.022^b^	5.425 ± 0.022^g^
100	17.770 ± 0.051^a^	4.902 ± 0.032^a^	22.671 ± 0.080^a^	3.625 ± 0.015^ab^	4.447 ± 0.018^a^	5.099 ± 0.022^i^
400	12.067 ± 0.015^h^	3.515 ± 0.013^e^	15.582 ± 0.027^g^	3.433 ± 0.008^e^	2.592 ± 0.004^i^	6.011 ± 0.019^c^
700	10.717 ± 0.034^i^	3.088 ± 0.014^f^	13.805 ± 0.029^h^	3.470 ± 0.023^de^	2.077 ± 0.003^j^	6.648 ± 0.013^a^
1000	8.912 ± 0.065^j^	2.440 ± 0.029^g^	11.351 ± 0.094^i^	3.653 ± 0.017^a^	1.841 ± 0.010^l^	6.167 ± 0.026^b^

*Chla* chlorophyll *a*, *Chlb* chlorophyll *b*, *Chla + Chlb* chlorophyll *a* + chlorophyll *b*, *Car* carotenoids, *Chla/Chlb* chlorophyll *a*/chlorophyll *b*, *Chl(a + b)/Car* (chlorophyll *a* + chlorophyll *b*)/carotenoids

Data are means ± SD from 3 independent experiments. Values denoted by the same letters in the columns do not differ statistically at *p <* 0.05

**Table 5:** Effect of [TBP][Br] on the photosynthetic pigment in seedlings of cucumber

Concentration of [TBP][Br] (mg kg^−1^ of soil DW)	Pigments (mg g^−1^ DW)					
	Chl*a*	Chl*b*	Chl*a* + Chl*b*	Chl*a*/Chl*b*	Car	Chl(*a* + *b*)/Car
10th day						
0	13.726 ± 0.002^g^	3.982 ± 0.020^e^	17.708 ± 0.017^h^	3.447 ± 0.018^d^	3.604 ± 0.004^f^	4.914 ± 0.010^h^
1	14.294 ± 0.056^f^	4.348 ± 0.004^d^	18.642 ± 0.052^f^	3.287 ± 0.016^fg^	3.984 ± 0.013^d^	4.679 ± 0.014^i^
10	15.308 ± 0.172^e^	4.469 ± 0.052^c^	19.777 ± 0.224^e^	3.425 ± 0.005^de^	3.895 ± 0.034^e^	5.077 ± 0.016^g^
100	13.137 ± 0.171^h^	3.829 ± 0.056^f^	16.966 ± 0.224^i^	3.431 ± 0.018^de^	3.429 ± 0.054^g^	4.948 ± 0.044^h^
400	8.014 ± 0.079^j^	2.242 ± 0.032^h^	10.256 ± 0.108^k^	3.575 ± 0.028^c^	1.961 ± 0.009^i^	5.230 ± 0.031^f^
700	4.019 ± 0.009^l^	0.984 ± 0.016^j^	5.003 ± 0.011^m^	4.085 ± 0.075^b^	0.931 ± 0.006^l^	5.376 ± 0.045^e^
1000	3.355 ± 0.011^m^	0.798 ± 0.008^k^	4.153 ± 0.010^n^	4.203 ± 0.049^a^	0.853 ± 0.005^m^	4.871 ± 0.035^h^
20th day						
0	17.427 ± 0.050^d^	5.347 ± 0.013^b^	22.724 ± 0.047^d^	3.260 ± 0.014^g^	4.145 ± 0.023^c^	5.494 ± 0.023^d^
1	18.804 ± 0.042^b^	5.699 ± 0.040^a^	24.502 ± 0.033^b^	3.300 ± 0.029^fg^	4.442 ± 0.029^a^	5.516 ± 0.035^d^
10	18.227 ± 0.063^c^	5.431 ± 0.025^b^	23.659 ± 0.066^c^	3.356 ± 0.020^ef^	4.292 ± 0.032^b^	5.512 ± 0.038^d^
100	19.087 ± 0.051^a^	5.774 ± 0.038^a^	24.862 ± 0.080^a^	3.306 ± 0.017^fg^	4.313 ± 0.022^b^	5.765 ± 0.029^c^
400	13.774 ± 0.039^g^	4.380 ± 0.019^d^	18.154 ± 0.058^g^	3.145 ± 0.005^h^	2.923 ± 0.007^h^	6.210 ± 0.020^b^
700	8.448 ± 0.020^i^	2.482 ± 0.021^g^	10.930 ± 0.039^j^	3.404 ± 0.022^de^	1.772 ± 0.003^j^	6.170 ± 0.033^b^
1000	6.606 ± 0.031^k^	1.910 ± 0.006^i^	8.516 ± 0.026^l^	3.459 ± 0.027^d^	1.310 ± 0.005^k^	6.503 ± 0.028^a^

*Chla* chlorophyll *a*, *Chlb* chlorophyll *b*, *Chla + Chlb* chlorophyll *a* + chlorophyll *b*, *Car* carotenoids, *Chla/Chlb* chlorophyll *a*/chlorophyll *b*,*Chl(a + b)/Car* (chlorophyll *a* + chlorophyll *b*)/carotenoids. Data are means ± SD from 3 independent experiments. Values denoted by the same letters in the columns do not differ statistically at *p <* 0.05

Both salts used in the study led to a systematic decrease in the content of Chl*a*, Chl*b*, Chl*a* + Chl*b* and carotenoids in cucumber leaves. The observed decreases in the level of these pigments after the application of the highest ILs concentrations reached in the case of [TBA][Br] 100% in relation to control and after the application of [TBP][Br] were even greater and amounted to over 300%. The observed decrease in the content of assimilative pigments in cucumber leaves was practically linearly correlated with an increase in the concentration of these salts in the soil and the observed inhibition of growth and decrease in the yield of cucumber fresh weight.

In addition to changes in the level of assimilation pigments, the Chl*a*/Chl*b* and Chl(*a + b*)/Car ratios are used to assess the physiological state of the plants. The increase in the value of Chl(*a + b*)/Car shows clearly the occurrence of oxidative stress in plants, as well as the decrease in the value of the Chl(*a + b*)/Car ratio. A decrease in the Chl(*a + b*)/Car value additionally indicates the plant organism’s antioxidant defence by increasing the carotenoid content, as these pigments are effective ROS sweepers and protect PSI and PSII photosystems (Chen et al. 2014; Wang et al. 2009; Zhang et al. 2013). After application of [TBA][Br], no major changes in Chla/Chlb values were found, regardless of the test date. On the other hand, in the case of [TBP][Br], there was a slight increase in the value of this biomarker, especially in the first research date, which indicates that the presence of this compound in the soil was a clear stress factor for cucumber plants. Both applied salts, on the other hand, led to an increase in Chl(*a + b*)/Car values, which was correlated with an increase in ILs concentration (Tables 4 and 5). Such a trend of changes in this indicator may indicate a situation in which plants have not increased their carotenoid content and thus have not yet undertaken an antioxidant defence.

### Effect of ILs on H_2_O_2_ content

In conditions of oxidative stress in plants, an increased overproduction of ROS can be observed, whose antioxidative systems cannot be detoxified. Among several types of ROS, the production and accumulation of H_2_O_2_ in the plant are quite special. Under oxidative equilibrium conditions, H_2_O_2_ is used by the plant as a physiologically relevant signal molecule; therefore, its content in plant cells never reaches 0. A sudden increase in the content of this ROS, on the other hand, is irrefutable evidence of the occurrence of oxidative stress in the plant, as it indicates an increase in the detoxification of superoxide anion by superoxide dismutase or indicates a lack of capacity of plant enzymatic detoxification mechanisms. At the same time, it should be remembered that H_2_O_2_ is one of the most stable ROS molecules, which diffuses freely through biological membranes, thus increasing its oxidative range (Demidchik 2015; Di Baccio et al. 2017; Kumar et al. 2013; Sánchez-Rodríguez et al. 2010).

The analysis of the results of the studies concerning the determination of the effect of [TBA][Br][Br] and [TBP][Br] on the amount of oxidative stress in cucumber seedlings showed that both fluids used caused H_2_O_2_ accumulation in vegetable cells (Table 6).

**Table 6:** Effect of ILs on the content of hydrogen peroxide (H_2_O_2_), malondialdehyde (MDA) and free proline in seedlings of cucumber

Concentration of ILs (mg kg^−1^ of soil DW)	MDA (μg g^−1^ FW)		H_2_O_2_ (μg g^−1^ FW)		Proline (mg g^−1^ FW)	
	[TBA][Br]	[TBP][Br]	[TBA][Br]	[TBP][Br]	[TBA][Br]	[TBP][Br]
10th day						
0	7.086 ± 0.870^ij^	11.437 ± 0.628^hi^	3.370 ± 0.136^f^	4.055 ± 0.068^j^	6.216 ± 0.195^g^	6.453 ± 0.365^j^
1	6.821 ± 0.137^j^	13.370 ± 1.109^gh^	3.653 ± 0.304^f^	5.609 ± 0.697^i^	5.111 ± 0.987^g^	6.538 ± 0.858^j^
10	6.892 ± 1.108^j^	15.327 ± 0.703^g^	3.723 ± 0.589^f^	6.380 ± 0.294^i^	6.264 ± 0.403^g^	7.732 ± 0.754^ij^
100	11.088 ± 0.924^gh^	18.487 ± 0.904^f^	5.524 ± 0.240^e^	12.664 ± 0.429^h^	9.753 ± 0.860^g^	41.457 ± 2.909^h^
400	20.973 ± 1.499^de^	33.062 ± 1.306^d^	6.341 ± 0.172^e^	17.925 ± 0.452^e^	42.569 ± 1.279^e^	74.125 ± 1.297^f^
700	28.581 ± 1.068^b^	41.001 ± 0.641^c^	8.616 ± 0.184^d^	20.256 ± 0.058^d^	76.747 ± 3.696^c^	96.197 ± 2.235^d^
1000	40.576 ± 0.573^a^	53.014 ± 0.493^a^	10.306 ± 0.121^c^	27.101 ± 0.900^b^	77.713 ± 4.912^c^	118.214 ± 1.123^c^
20th day						
0	9.158 ± 0.348^hi^	8.368 ± 0.563^j^	10.510 ± 0.354^c^	12.080 ± 0.283^h^	7.644 ± 0.365^g^	9.997 ± 0.953i^j^
1	9.196 ± 0.181^i^	9.962 ± 0.412^ij^	11.840 ± 0.118^b^	12.608 ± 0.196^h^	7.194 ± 0.572^g^	8.643 ± 0.789^ij^
10	11.832 ± 0.180^fg^	11.268 ± 0.117^hi^	11.911 ± 0.204^b^	14.421 ± 0.068^g^	9.135 ± 0.521^g^	11.417 ± 1.345^i^
100	13.421 ± 0.244^f^	12.194 ± 0.316^h^	11.570 ± 0.392^b^	16.455 ± 0.207^f^	28.097 ± 1.743^f^	53.897 ± 1.700^g^
400	20.308 ± 0.730^e^	22.075 ± 0.749^e^	13.929 ± 0.320^a^	18.718 ± 0.139^e^	51.839 ± 2.325^d^	83.007 ± 2.165^e^
700	22.967 ± 0.121^cd^	34.459 ± 0.936^d^	12.048 ± 0.070^b^	23.464 ± 0.620^c^	117.775 ± 5.135^b^	155.928 ± 1.586^b^
1000	23.866 ± 0.491^c^	46.386 ± 0.684^b^	11.854 ± 0.614^b^	29.834 ± 0.139^a^	128.676 ± 5.369^a^	175.624 ± 0.576^a^

Data are means ± SD from 3 independent experiments. Values denoted by the same letters in the columns do not differ statistically at *p <* 0.05

Salt containing phosphorus in the cation was more toxic, and the observed increase in H_2_O_2_ concentration was positively correlated with the applied concentration. After the introduction of [TBP][Br] into the substrate in the highest concentrations (700–1000 mg kg^−1^ of soil DW), a large increase in H_2_O_2_ content was observed, which amounted to about 500–650% in the first analysis period and 190–250% in the second period, respectively, compared with the control. In the case of ammonium salt, however, a large increase in H_2_O_2_ levels occurred only on the 10th day after the introduction of IL to the substrate and at the highest concentrations was about 250–300% compared with the control. In the second test period, changes in the H_2_O_2_ content of cucumber leaves were no longer so pronounced. This observed trend of decrease of H_2_O_2_ accumulation in cucumbers together with the date of analysis indicates the fact that the plant has activated enzymatic sweepers of this ROS. A similar increase in H_2_O_2_ content in plants affected by ILs was also observed by Zhang et al. (2013) for duckweed, Cvjetko Bubalo et al. (2014) for barley and Biczak et al. (2017) for common radish. However, there are studies (Biczak 2017; Pawłowska et al. 2019b) treating the decrease of H_2_O_2_ level in plants grown on IL medium; therefore, it should be assumed that the direction of changes in the content of this ROS is highly dependent on the plant species characteristics, type of compound and applied concentration, as well as on the time of plant exposure to ILs.

### Effect of ILs on MDA content

In addition to hydrogen peroxide, changes in MDA levels in cucumber leaves were also determined in the presented studies (Table 6). Analogously to the changes in H_2_O_2_ content, the level of MDA increased successively with the concentration of the examined ILs in the soil. Tetrabutylammonium bromide in the first term of analysis led to about 500% increase in MDA content in plants grown on the soil with the highest concentration of this salt (1000 mg kg^−1^ of soil DW), while in the second term of analysis, such increase was already less than 250%. The situation was different after applying [TBP][Br]. The highest concentration of this IL on the 10th day after its introduction into the soil led to an approximately 450% increase in the MDA content of cucumber, and in the next analysis period, an even higher accumulation of MDA was observed, reaching already about 550% compared with the control. This direction of change in the MDA level makes it possible to conclude that antioxidant systems in cucumber after application of [TBA][Br] start to cope with stress after a shorter time than in plants exposed to [TBP][Br]. Similar conclusions were also reached by Deng et al. (2017), Cvjetko Bubalo et al. (2014), Liu et al. (2015, 2016a, b, 2018a, b), Pawłowska et al. (2019b) and Xu et al. (2018), and the observed trend of changes in MDA levels is explained by the fact that high concentrations of ILs generate such a high degree of oxidative stress, which the plant’s defence mechanisms are no longer able to cope with. Under these conditions, ROS oxidize the fatty acids present in the biological membranes, resulting in large quantities of MDA.

### Effect of ILs on free proline content

According to the available literature, proline is an amino acid which reacts with singlet oxygen and hydroxyl radical to produce less reactive products. Anjaneyulu et al. (2014) and Liu et al. (2014) further claim that proline regulates the osmotic potential of plant cells, reduced under oxidative stress conditions, and can therefore be considered to be an antioxidant chemical. In opposition to the above statements, there are results of a study by Sánchez-Rodríguez et al. (2010), who believe that the increase in proline content is only a biomarker of oxidative stress in plants and that proline itself has no proven antioxidative properties.

As a result of the conducted studies, a significant huge increase in free proline content in cucumber seedlings was observed, starting from the objects on which the tested ILs were applied at a concentration of 100 mg kg^−1^ of soil DW (Table 6). Similarly, as it was observed in the case of MDA and H_2_O_2_ cucumber leaf accumulation, greater changes in free proline levels were found in plants grown on medium containing [TBA][Br]. Regardless of the date of analysis, the accumulation of proline reached as much as about 1800% after applying the highest concentration of this salt. Under analogous cultivation conditions, in objects with [TBA][Br], the increase in free proline level was from 1200 to 1600%, respectively, for the first and second test dates. The results of the study are confirmed by numerous literature reports, which report on the increase in free proline content in plants treated with ILs. As a rule, the increase in free proline levels observed and described in the literature was strictly dependent on the concentration used (Liu et al. 2014, 2018a; Pawłowska et al. 2019b; Xu et al. 2018).

### Effect of ILs on antioxidant enzyme activities

In addition to low-molecular antioxidants like ascorbic acid, flavonoids and tocopherol, a whole range of enzymes are present in plant organisms to detoxify ROS. The following should be mentioned here: superoxide dismutase (SOD), peroxidase (POD), catalysis (CAT) or glutathione reductase (GR). The action of these enzymes is specialized and combined in a whole sequence of chemical reactions because the product of one of these enzymes immediately becomes an activator and a substrate for the next enzyme. This sequence of reactions continues until non-toxic particles are formed from ROS (Sánchez- Rodríguez et al. 2010; Gengmao et al. 2015).

The family of metalloenzymes, generally referred to as SOD, is responsible for the decomposition of superoxide radicals into H_2_O_2_ and O_2_. This important transformation determines that the activity of SOD is always determined in works describing the activity of the antioxidant mechanism in plants subjected to various stress factors (Biczak 2017; Chen et al. 2014, 2018, 2019; Fan et al. 2019; Liu et al. 2014, 2016a and b; Zhang et al. 2013). However, the analysis of the results presented in the available literature does not allow to draw clear conclusions as to the direction of changes in SOD activity in plants for which the presence of ILs in their growth environment is an abiotic stress factor. Some authors (Cvjetko Bubalo et al. 2014; Liu et al. 2014, 2018a; Xu et al. 2018, 2020) found an increase in SOD activity under the influence of ionic liquids present in the substrate barley seedlings, wheat seedlings and broad bean plants, respectively, while Chen et al. (2018) showed a decrease in SOD activity in wheat seedlings, which would be correlated with the concentration of these salts in the substrate.

The analysis of own research results shows that the activity of superoxide dismutase depended on the compound used (Table 7). After the application of [TBA][Br], a slight systematic decrease in SOD activity can be observed in the first analysis date, while in the second analysis date, no statistically proven differences between SOD activity in IL-affected and control objects were found. In the case of [TBP][Br], a decrease in SOD activity was found at both test dates, starting from the concentration of 10 mg kg^−1^ of soil DW, and for the highest of the applied concentrations, it was about 50% at the first date and about 25% at the second date of analysis, respectively. Such changes in SOD activity are confirmed in the works of Biczak et al. (2017), Fan et al. (2019) and Liu et al. (2018b), in which after applying low concentrations, an increase in SOD activity was observed, while high concentrations led to a clear inhibition of this enzyme activity. The authors explain this trend of SOD activity changes by the fact that the observed initial increase in SOD activity is a clear indication of ongoing antioxidant defence, while the observed decrease in SOD activity found at a high concentration of ILs indicates large damage to plant cells, effectively preventing the activation of more antioxidant enzymes.

**Table 7:** Changes in enzymatic activities of superoxide dismutase (SOD), peroxidase (POD) and catalase (CAT) in seedlings of cucumber treated with ILs

Concentration of ILs (mg kg^−1^ of soil DW)	The activity of enzymes					
	SOD (U mg^−1^ protein)		POD (U mg^−1^ protein min^−1^)		CAT (U mg^−1^ protein min^−1^)	
	[TBA][Br]	[TBP][Br]	[TBA][Br]	[TBP][Br]	[TBA][Br]	[TBP][Br]
10th day						
0	6.581 ± 0.596^a^	6.294 ± 0.291^ab^	1.280 ± 0.053^g^	1.707 ± 0.015^i^	0.0352 ± 0.0025^gh^	0.0390 ± 0.0015^f^
1	6.277 ± 0.854^ab^	7.260 ± 0.817^a^	0.994 ± 0.019^g^	1.326 ± 0.038^i^	0.0406 ± 0.0010^g^	0.0393 ± 0.0010^f^
10	5.814 ± 0.288^ab^	5.272 ± 1.019^bcde^	1.184 ± 0.057^g^	1.735 ± 0.078^i^	0.0377 ± 0.0025^g^	0.0345 ± 0.0044^f^
100	5.519 ± 0.294^abc^	4.530 ± 0.260^def^	2.074 ± 0.010^f^	2.659 ± 0.027^h^	0.0696 ± 0.0033^f^	0.0648 ± 0.0013^e^
400	5.257 ± 0.091^abc^	4.226 ± 0.665^ef^	2.722 ± 0.168^f^	3.554 ± 0.057^g^	0.1056 ± 0.0032^d^	0.1472 ± 0.0021^c^
700	4.389 ± 0.389^c^	4.258 ± 0.347^ef^	3.528 ± 0.092^e^	4.997 ± 0.108^f^	0.1609 ± 0.0028^b^	0.1761 ± 0.0011^a^
1000	4.297 ± 0.194^c^	3.161 ± 0.508^f^	7.521 ± 0.284^d^	9.322 ± 0.255^d^	0.1880 ± 0.0018^a^	0.1727 ± 0.0038^a^
20th day						
0	5.568 ± 0.528^abc^	6.112 ± 0.217^abc^	2.348 ± 0.101^f^	2.433 ± 0.043^h^	0.0262 ± 0.0083^h^	0.0214 ± 0.0018^h^
1	6.307 ± 0.810^ab^	5.909 ± 0.515^abcd^	2.252 ± 0.059^f^	2.528 ± 0.059^h^	0.0380 ± 0.0048^g^	0.0262 ± 0.0015^gh^
10	5.391 ± 0.450^abc^	5.201 ± 0.208^bcde^	2.310 ± 0.094^f^	2.736 ± 0.115^h^	0.0457 ± 0.0018^g^	0.0322 ± 0.0033^fg^
100	5.378 ± 0.192^abc^	5.126 ± 0.027^bcde^	2.469 ± 0.143^f^	6.695 ± 0.028^e^	0.0737 ± 0.0035^f^	0.0385 ± 0.0014^f^
400	5.785 ± 0.078^ab^	5.448 ± 0.654^bcde^	9.114 ± 0.379^c^	11.028 ± 0.192^c^	0.0922 ± 0.0029^e^	0.0769 ± 0.0031^d^
700	5.000 ± 0.374^bc^	5.049 ± 0.135^bcde^	11.705 ± 0.798^b^	16.219 ± 0.618^b^	0.1239 ± 0.0033^c^	0.1408 ± 0.0035^c^
1000	5.325 ± 0.530^abc^	4.690 ± 0.175^cde^	14.914 ± 0.115^a^	18.692 ± 0.267^a^	0.1186 ± 0.0060^c^	0.1604 ± 0.0020^b^

Data are means ± SD from 3 independent experiments. Values denoted by the same letters in the columns do not differ statistically at *p <* 0.05

Two enzymes, catalase (CAT) and peroxidase (POD), are responsible for the removal of H_2_O_2_ resulting from the dismutation of the superoxide anion. Although catalase is considered to be the primary enzyme responsible for the decomposition and removal of H_2_O_2_ from plant cells, the data presented in the literature, describing the changes in the activity of this enzyme resulting from the introduction of ILs into the substrate, do not allow to clearly determine the direction of such changes. There are studies that have observed a significant decrease in catalase activity in plants treated with ILs (Biczak 2017; Chen et al. 2018; Liu et al. 2014, 2016b). However, Cvjetko Bubalo et al. (2014), Fan et al. (2019), Gengmao et al. (2015), Liu et al. (2018a) and Xu et al. (2018) prove that the reaction of plants to stress caused by the presence of ILs in the environment is always an increase in CAT activity. In cucumber seedlings, we can also observe a systematic increase in catalase activity, which, for both compounds and at both test dates, was clearly visible from the concentration of 100 mg kg^−1^ of soil DW. In the case of [TBA][Br] for the concentration of 1000 mg kg^−1^ of soil DW at both analysis dates, the catalase activity increased by about 450%, similarly as for [TBP][Br] at the first date of the study, while at day 20 of the introduction of this salt into the soil, the CAT activity was already over 600% higher in relation to control (Table 7). Such enormous increases in CAT activity are fully justified in relation to H_2_O_2_ accumulation in cucumber leaves, as described in “Effect of ILs on H_2_O_2_ content”.

An analogous direction of change with catalase can be observed for peroxidase (POD). And for this enzyme, starting from the concentration of 100 mg kg^−1^ of soil DW, an almost linear increase in activity positively correlated with the concentration of ILS in the soil can be observed (Table 7). In the first term of the study, the increase in peroxidase activity from objects with the highest concentration of both ILs was about 450% compared with the control. However, in the second period of analysis, the concentration of 1000 mg kg^−1^ of soil DW in the case of [TBA][Br] caused an increase in peroxidase activity by about 530%, and for [TBP][Br], the increase in activity of this enzyme was even greater and amounted to about 650%. It is worth noting that on day 20, since the introduction of ILs to the soil, the POD activity was higher in all objects, as compared with the first research date. The observed changes in POD activity combined with a decrease in chlorophyll content, according to some authors (Herman et al. 1998), may indicate premature ageing of plants grown under stress conditions. However, such a huge increase in POD activity is not entirely beneficial for plants, because this enzyme also removes H_2_O_2_ signal molecules from the body, which adversely affects their metabolism (Dragišić Maksimović et al. 2013; Wang et al. 2009). Despite this, the increase in POD activity is regarded as the most reliable biomarker of oxidative stress, because regardless of its cause, the activity of this enzyme always increases under conditions of H_2_O_2_ overproduction (Anjaneyulu et al. 2014; Biczak 2017; Biczak et al. 2017; Liu et al. 2014, 2016b; Pawłowska et al. 2019b; Xu et al. 2018).

### Interactions between tested parameters

Using the analysis of the effect of agent *η*^2^, carried out by the method of variance analysis, the percentages of all variable factors influencing the determined parameters determining phytotoxicity and oxidative stress level in cucumber seedlings were determined and presented in Tables 8 and 9.

**Table 8:** Participation of variable factors in the formation of assayed plant parameters (%) treated with [TBA][Br]

Parameter	Term (A)	Dosage of ILs (B)	A × B	Error
POD	17.763	66.910	14.075	0.253
CAT	3.106	89.837	6.638	0.419
SOD	0.356*	54.448	18.726	26.470
MDA	0.696	86.149	12.766	0.389
H_2_O_2_	71.256	16.083	12.261	0.400
Proline	5.272	87.192	7.290	0.246
Chl *a*	0.366	91.990	7.623	0.020
Chl *b*	0.599	90.595	8.712	0.093
Chl *a* + Chl *b*	0.413	91.713	7.848	0.026
Car	0.294	95.033	4.654	0.020
Dry weight	0.307	97.101	1.484	1.108
Yield	8.634	83.058	7.709	0.599

*Statistically insignificant at *p* < 0.05

**Table 9:** Participation of variable factors in the formation of assayed plant parameters (%) treated with [TBP][Br]

Parameter	Term (A)	Dosage of ILs (B)	A × B	Error
POD	21.153	65.052	13.699	0.096
CAT	4.639	92.100	3.128	0.123
SOD	2.881	64.302	18.100	14.716
MDA	4.069	95.003	0.756	0.172
H_2_O_2_	10.163	86.547	3.090	0.200
Proline	3.514	91.916	4.523	0.047
Chl *a*	17.431	81.473	1.081	0.014
Chl *b*	20.114	78.445	1.419	0.021
Chl *a* + Chl *b*	18.071	80.775	1.141	0.014
Car	6.275	92.981	0.723	0.021
Dry weight	1.661	93.352	4.599	0.388
Yield	23.198	71.51	4.862	0.379

The analysis of the data contained in these tables makes it possible to unequivocally state that in the case of [TBP][Br], all the changes in the biomarkers of phytotoxicity and oxidative stress parameters determined resulted from the increase in the concentration of this salt in the soil. Analogous conclusions can also be drawn from the results obtained after the application of [TBA][Br], with the only difference that the changes in H_2_O_2_ content were more influenced by the time of plants’ exposure to the tested compound (test date). Due to very high error values, it was not possible to determine which of the analysed factors (test date or ILs concentration) had a greater effect on SOD activity conversions.

Using the analysis of the effect of agent *η*^2^, based on the three-factor analysis of variance, an attempt was also made to answer the question whether the genus ILs had a significant effect on changes in the determined phytotoxicity indices and biomarkers of oxidative stress in cucumber plants (Table 10).

**Table 10:** Participation of variable factors in the formation of assayed plant parameters (%)

Parameter	Term (A)	ILs (B)	A × B	Dosage of ILs (C)	A × C	B × C	A × B × C	Error
POD	22.812	2.840	0.577	59.812	12.407	1.126	0.537	0.166
CAT	4.638	0.038	0.123	88.530	2.423	1.467	2.530	0.250
SOD	5.189	6.321	2.816	47.015	15.729	4.819	0.488	17.624
MDA	2.861	8.337	0.585	79.328	2.504	4.529	1.811	0.225
H_2_O_2_	12.940	31.594	0.185	35.726	2.463	15.950	0.983	0.159
Proline	5.268	4.045	0.001*	83.273	5.353	1.707	0.219	0.135
Chl*a*	5.892	3.229	5.558	80.189	1.163	2.842	1.113	0.015
Chl*b*	7.319	1.273	7.730	76.664	1.210	4.178	1.591	0.035
Chl*a* + Chl*b*	6.223	2.700	6.043	79.505	1.168	3.125	1.218	0.016
Car	2.246	1.767	2.136	90.446	0.800	1.951	0.634	0.019
Dry weight	0.005*	0.130	0.093	97.917	0.409	0.307	0.225*	0.913
Yield	6.181	3.845	0.085	79.399	7.410	2.234	0.177	0.669

The analysis showed that only changes in the level of H_2_O_2_ in cucumber plants depended on the type of ILs used, and the calculated percentage of this factor can be compared, as to the magnitude of the effect, with an increase in the concentration of the examined salts in the soil. The three-factor analysis confirmed that the remaining indicators of both stress and phytotoxicity were most affected by the concentration of both compounds.

**Fig. 4:**
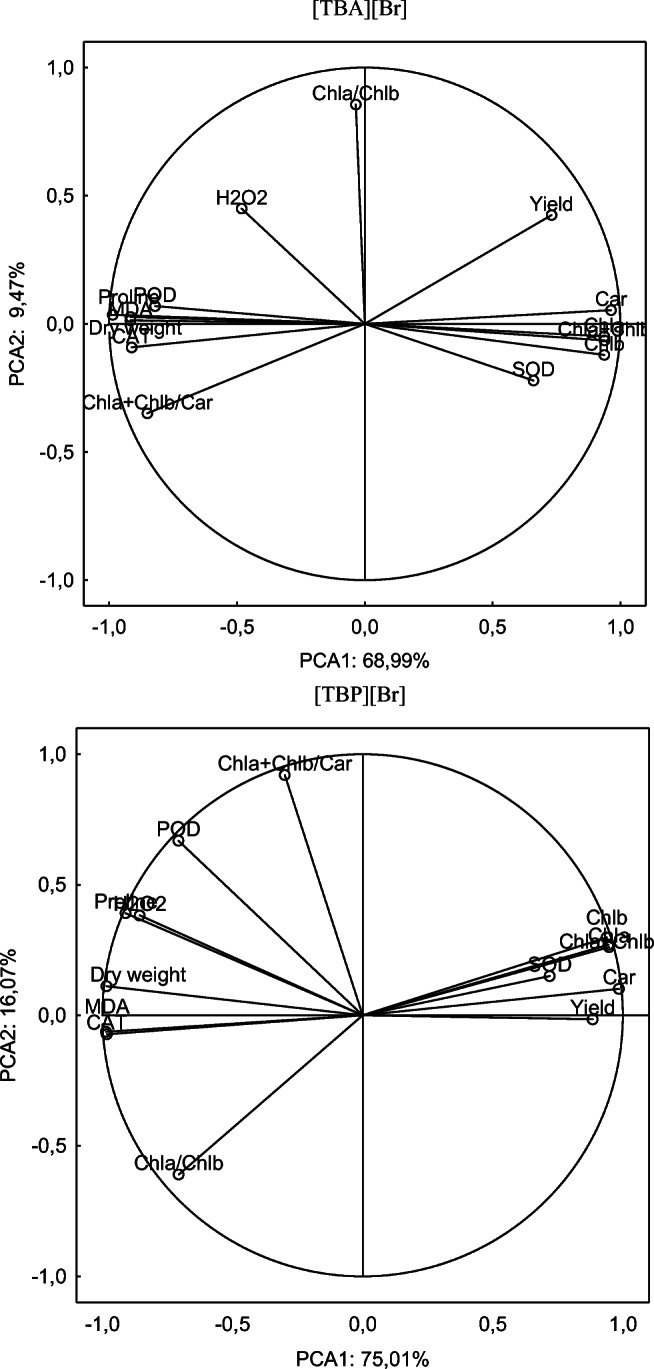
Analysis of the main components of the impact of ILs on cucumber seedlings, depending on the type of ILs

In order to establish relationships between individual phytotoxicity and oxidative stress parameters in cucumber seedlings subjected to ILs, the analysis of the main components (PCA) was also performed. The obtained results are presented in Fig. 4, which also contains the Pearson correlation coefficients between yield, pigment content, MDA, H_2_O_2_, proline and dry weight, SOD, CAT and POD activity in cucumber growing on soil with the addition of [TBA][Br] and [TBP][Br].

The analyses carried out indicate that for both compounds used, only the carotenoid content shows a positive relation to both the PCA1 and PCA2 axes and a negative correlation to both axes shows CAT activity. POD activity, proline, H_2_O_2_ and dry weight content show a negative relationship with PCA1 and a positive relationship with PCA2. However, no common phytotoxicity and oxidative stress indicator were found to show a positive correlation with PCA1 and a negative correlation with PCA2. The distribution of vectors around the axis for [TBA][Br], which consists of the first two factors, describes a value of 78.46% (PCA1 68.99%, PCA2 9.47%), while for [TBP][Br], the distribution of vectors around the axis was 91.08% (PCA1 75.01%, PCA2 16.07).

## Conclusion

The studies carried out to determine the effect of [TBA][Br][Br] and [TBP][Br] on the growth and development of early stages of cucumber showed that the applied ILs were characterized by phytotoxicity, the size of which depended mainly on the applied concentration of these salts. This can be proved by the size of the inhibition of the length of aboveground parts of plants and their roots, the yield of fresh weight and the calculated values of EC_50_. Statistical analysis of the results obtained proved, however, that there are no statistically justified differences in phytotoxic effects between the fluids used.

The applied ILs led to a decrease in the content of assimilative pigments in cucumber seedlings, which resulted in a decrease in the yield of cucumber plants. [TBA][Br] and [TBP][Br] also led to oxidative stress in cucumber seedlings, which was indicated by high accumulation of MDA, H_2_O_2_ and free proline in leaves of this plant. In response to the oxidative stress, the plants launched a system of antioxidative enzymes, which was reflected in the observed increase in POD and CAT activity. Statistical analysis to determine and compare the degree of influence of variable factors (concentration and term of analysis) showed that after applying both ILs, changes in all oxidative stress and phytotoxicity parameters mainly depended on the concentration of these salts. It should be noted, however, that in terms of numbers, greater changes in the content of the examined oxidative stress indices and activity of antioxidative enzymes were observed after introduction of [TBP][Br]. However, the analysis of the effect of agent *η*^2^ based on the three-factor analysis of variance proved that ILs genus did not have a significant influence on the direction of changes in the determined phytotoxicity indices and biomarkers of oxidative stress in cucumber plants.
